# Prisoner's science

**DOI:** 10.1128/mbio.02633-23

**Published:** 2023-12-20

**Authors:** Arturo Casadevall, Ferric Fang

**Affiliations:** 1 Department of Molecular Microbiology and Immunology, Johns Hopkins School of Public Health, Baltimore, Maryland, USA; 2 Departments of Laboratory Medicine, Pathology and Microbiology, University of Washington, Seattle, Washington, USA; Duke University, Durham, North Carolina, USA

**Keywords:** ethics, sociology, game theory

## Abstract

Decisions involving cooperation or competition are common in science. Here, we consider three situations frequently encountered in the biomedical sciences, namely, establishing priority, sharing reagents, and selecting a journal for publication, through the lens of the prisoner's dilemma. In each situation, cooperation is the best strategy for scientists and for science.

## OPINION/HYPOTHESIS

*The best way to keep a prisoner from escaping is to make sure he never knows he is in a prison*.

-Fyodor Dostoevsky

Scientists often face decisions in which they must choose between cooperation and competition. Cooperation with other scientists may lead to better science, as ideas can be vetted more thoroughly through discussions, and projects are enhanced by the different perspectives of cooperating research groups. However, the economics of science, in which prestige accumulates according to the priority rule, dictates that credit is given to the individual or group that is first in discovery (1). This creates a winner-takes all-outcome, which discourages cooperation that could lead to the dilution of credit (2). Since resources for scientific work such as positions, grants, trainees, and laboratory space are allocated based on credit, the choice for scientists between cooperation and competition becomes a problem in game theory.

We have previously extolled the virtues of cooperation and argued for fundamental changes to the economics of science, including abandonment of the priority rule. In this essay, we explore the choices of scientists viewed as a simple game theory problem, the prisoner's dilemma. First proposed in 1950 by Merrill Flood and Melvin Dresher of the RAND Corporation as a model for analyzing cooperation (3), and subsequently adapted to a prison setting by Albert Tucker (4), the prisoner's dilemma has been the subject of exhaustive study and applied to numerous problems in economics and social sciences. While it is not our goal to carry out a detailed analysis here, we would like to use the framework of the prisoner's dilemma to show its general applicability to the choices made by scientists, in the hope of stimulating future studies of the application of game theory to understanding the sociology of science.

### The prisoner's dilemma

The prisoner's dilemma is essentially some variation of the following hypothetical scenario: two criminals, let us call them Ferric and Arturo, decide to steal a motorcycle and go for a joyride. The police arrest them and place them in separate cells. Each is told that they face grand larceny charges that carry 10 years in prison, but if they confess and agree to provide evidence against the other, the sentence will be reduced to 5 years. However, if one chooses to remain silent and the other confesses, the silent prisoner will get 10 years in jail. What Ferric and Arturo do not know is that the police do not have enough evidence for a conviction. Therefore, if both remain silent, both will be released. Ferric and Arturo face the choice of cooperating with each other by remaining silent or defecting by admitting their role and providing evidence against the other. The decision whether to cooperate or defect must be made with incomplete information, since neither knows what the police know, and the two are unable to communicate with each other. Faced with these choices, the rational self-interested choice is to defect and settle for 5 years in jail, rather than take the risk of serving a longer sentence. However, if both men were to cooperate by remaining silent, each would walk free, illustrating how individuals acting in their own self-interest do not always produce an optimal collective outcome.

The prisoner's dilemma and its many permutations have been studied extensively in a variety of fields including economics, international relations, psychology, sociology, animal behavior, and environmental science (5). The profound philosophical and ethical implications of the prisoner's dilemma have been used to support Kant's categorical imperative, his central principle of moral behavior (6), which translates as “Act only according to that maxim whereby you can at the same time will that it should become a universal law” (7). The literature on this game is now enormous, with the keyword “prisoner's dilemma” yielding more than 170,000 articles in Google Scholar. Here, we consider three situations commonly encountered by biomedical scientists as framed by the prisoner's dilemma, in which defection may lead to the greatest gain in the short run, but cooperation has tremendous advantages in the long run, making it the preferred strategy (Table 1).

**TABLE 1:** Three common situations faced by scientists and the choices to cooperate or defect

Situation	Cooperation	Defection
Fear of being scooped	Work with competing group	Attempt to publish first
Sharing reagents	Negotiate sharing agreement	Refuse to share
Choosing a journal	Choose a journal most appropriate for content	Choose the journal with the highest impact factor

### Scientific prisons

#### Fear of being scooped

Since credit in science is based on the priority rule, the first to report a discovery gets all the credit (8). We have referred to the fear of being scooped as “gelatophobia” (9). When scientists make a discovery, one of their immediate concerns is whether other groups are on the verge of reporting a similar discovery. Since scientists have incomplete knowledge about the status of competing groups, they must make their choices in a version of the prisoner's dilemma, deciding between defection or cooperation without the benefit of complete information. Defection in this instance might consist of rushing to finish the work so that it can be published before any potential competitors, which would monopolize the credit, according to the priority rule. However, defection carries the risk that the competing group may have already submitted a manuscript describing the finding, which has been accepted and is awaiting publication. The scientist's dilemma is that attempting to obtain all the credit runs the risk of receiving no credit at all. A more cooperative alternative might be to contact the rival group and offer to coordinate publication, hoping to share credit. However, this approach could dilute the amount of prestige associated with the discovery. A scientist might also publish the finding in a preprint, in the hope of establishing priority, but this strategy also entails some risk, as competitors might see the preprint and expedite the publication of a competing work. Yet another cooperative strategy might involve the competing groups bundling their findings into a single paper that would be stronger than separate manuscripts from individual laboratories. Unfortunately, the current economics of science that depend on the priority rule and the disproportionate credit assigned to first authors, along with the need for first-author papers to secure jobs and grants, would tend to discourage such an option (10).

Fear of being scooped also accompanies the presentation of unpublished data at a scientific meeting, where the presenter runs the risk that a competitor in the audience may use the presented information to hasten the publication of their own work and establish a claim of priority. For the presenting scientist, cooperation would involve sharing unpublished data so that other scientists are more rapidly aware of recent results, whereas defection would involve limiting the presentation to work that is already (or very nearly) published. Today, many scientists choose to defect, reducing the value of meetings, which all too frequently feature presentations of already published information. The net result is less communication and slower transmission of new scientific information.

#### Sharing reagents

Progress in biomedical science is often critically dependent on specialized reagents that are generated as part of the research process. When the existence of such reagents becomes known, the laboratory that created the reagent often receives requests to share it with other researchers, who may be competitors. Many journals require the sharing of reagents as a condition for publication, but there is no obligation to share reagents that have not yet been reported in a publication. When a request for an unpublished reagent is received, the scientist receiving the request faces a version of the prisoner's dilemma and must choose whether to share the reagent without the benefit of complete information about the request. The scientist does not know how the reagent will be used and risks sharing the reagent with competing groups who may be performing similar work. In some cases, this could result in scooping of the scientist's own research. In this dilemma, cooperation consists of sharing the reagent, while defection consists of ignoring the request or declining to share the reagent. A compromise position might be to offer to share the reagent while negotiating some limits to its use or by agreeing to share the credit for any resulting discoveries.

#### Choosing journals

In today's economics of science, scientists derive disproportionate benefit from publishing their work in highly selective journals with high impact factors. We have written extensively about this sociologically maladaptive phenomenon in the past and compared it to a form of mania (11, 12). We have further argued that impact factor mania is a version of Garrett Hardin's “tragedy of the commons” in which scientists act rationally in trying to get their papers into high impact journals but in doing so distort the value system of science and create perverse incentives (12). Like the prisoner's dilemma, the tragedy of commons involves a tension between actions that primarily benefit the individual versus more collective interests (13). Impact factor mania is primarily driven by scientists themselves, who seek the disproportionate rewards associated with publication in certain journals and persist in that behavior to the detriment of science (12). There are numerous ways by which impact factor mania damages science, including an emphasis of research impact over importance, incentivizing misconduct, and delaying the publication of new findings as scientists cascade their papers through journals hoping to maximize the impact factor. Viewing journal selection through the prism of the prisoner's dilemma, we see a cooperative choice as selecting the most appropriate journal for a given paper based on the scope of the journal and the expertise of the editors, whereas defection would be to seek a journal solely based on impact factor. By defecting, scientists are acting rationally in the sense that they are seeking the benefits that come with publication in journals with high impact factors, but unfortunately, this behavior is collectively detrimental to science. Today, the prevalence of impact factor mania among biomedical scientists suggests that most are choosing to defect. In deciding whether to cooperate or defect, the scientist must work with incomplete information, since the publication process is notoriously unpredictable and capricious. Opting for a high impact journal might mean months of effort and enduring many rejections, but selecting a more specialized journal does not necessarily make the process easier since such journals may still put the paper through rigorous review that identifies the need for more work before publication. Neither option guarantees rapid publication, creating the risk that the relevance of the work will wane or be superseded by the publication of other relevant results.

### How to get out of jail?

In the game *Monopoly*, players who are sent to jail may return the game if they are fortunate enough to have a “get-out-of-jail free” card. Although there is no such card in science, we suggest that the costs of cooperation are relatively minor in comparison to the costs of defection. This may explain why, in a variety of settings, subjects facing the prisoner's dilemma often appear to prefer mutual cooperation over defection, even though it initially seems more rational to defect (14). In one of the few studies of the prisoner's dilemma that has involved actual prisoners, German researchers found that more than half of inmates preferred to cooperate rather than defect (15). Why might this be the case? Perhaps it is because of social norms that discourage individuals from acting purely out of self-interest. Criminal societies are notoriously intolerant of informants, which provides a powerful deterrent to any prisoner thinking of defecting. Norms in criminal societies strongly discourage members from becoming “rats” or “stool pigeons.” Thus, there is a strong incentive for prisoners to choose the long-term benefits of cooperation, even when defection seems to offer better short-term prospects.

Cooperation is also the best option for scientists in the long run, and for somewhat analogous reasons. Like criminal enterprises, science operates within a set of norms that can negate the short-term benefits of defection in prisoner's dilemma scenarios. For scientists to remain in good standing with their colleagues, they must adhere to these norms, which carry significant penalties if violated.

Let us consider the potential benefits of cooperation to those that adhere to the norms of science. The scientist who fears being scooped might cooperate by reaching out to a competing lab to discuss their common interests and the possibility of simultaneous publication. A famous example of such cooperation is the discovery of the interstellar 21 cm hydrogen radio spectral line in 1951. Three groups in Australia, the Netherlands, and the United States were racing to detect the radio signature of interstellar hydrogen, which would give astronomers a new window for studying the universe. Initially, the Dutch group took the lead, but their progress was delayed by an equipment fire (16). Subsequently, the American team of Ewen and Purcell were the first to detect the hydrogen signal (17). They shared the news with the other groups and asked the journal *Nature* to delay publication until the Dutch and Australian researchers could confirm their findings. Eventually, the three groups published back-to-back reports in the same issue (18–20). This is a wonderful story of international cooperation. By informing the other groups of their progress, Ewen and Purcell hastened confirmation of the discovery. By arranging for simultaneous publication, they demonstrated their generosity, humanity, and recognition that priority was largely a matter of luck. One of us (A.C.) has taken a similar approach in recent years by simultaneously publishing with other groups who independently discovered the phenomena of fungal cell gigantism (21, 22), non-lytic fungal exocytosis (23, 24) and macrophage-to-macrophage transfer (25, 26), and can attest that friendship, increased trust, and cooperation between research groups can result when everyone wins. It is also important to remember that priority is not the only determinant of whether a publication is subsequently cited. Although citation rates are an imperfect indicator of article quality, the positive correlation between citation rates and independent measures of research quality (27) suggests that scientists are more likely to cite a work if it is of higher quality. It is more important to be good than first.

To scientists who are concerned about presenting their unpublished work at a scientific meeting, we argue that the best option is cooperation and the sharing of unpublished work, as this not only results in more rapid scientific communication but also affords an opportunity to receive critical input from colleagues that can improve the quality of research prior to publication. The benefit is maximized if all scientists agree to share. Scientists concerned about priority can always stake their claim by drafting a preliminary paper that is posted as a preprint.

When a scientist receives a request for an unpublished reagent, cooperation in the form of sharing is again the preferred option. It is often possible to share reagents as part of a collaboration, which benefits both laboratories who receive shared credit in resulting publications. Sharing is also good for science because it promotes research progress and provides independent corroboration of a reagent's utility and limitations. Furthermore, sharing avoids all the disadvantages of refusing to share, which can damage the reputation of scientists who choose to hoard resources.

We recognize the longstanding tension between competition and cooperation with regard to data sharing in science (28, 29). Although an open data policy can help to validate findings and expedite scientific progress, scientists remain concerned about losing credit for their work (30). A survey of geneticists found that many have experienced benefits from sharing research results in the form of new collaborative opportunities, publications, and funding, although a smaller percentage reported missing out on credit or opportunities to commercialize their research (31).

The selection of journals poses a more complex dilemma than scooping or reagent sharing because there are many journals to choose from, and the choice must take place in a maladaptive scientific culture that defies reason. Nevertheless, many scientists, especially younger ones, feel like prisoners in an impact factor game that obligates their participation due to the disproportionate rewards for success (32). Given this difficult situation, we can only advise scientists to resist the temptation of judging work based on the prestige of a journal and to take solace in the fact that good science rises to the top no matter where it is published. Journal impact factor is in fact a poor predictor of the quality of peer review or the citation rate of individual papers (33, 34), and high impact journals have some of the highest retraction rates (35). Some of the most important scientific papers in history were published in venues that today may be less well known to scientists in the biomedical sciences, such as Griffith's discovery of the transforming principle (*Journal of Hygiene*, reference 36), Krebs' discovery of the tricarboxylic acid cycle (*Enzymologia*, reference 37), Glick's demonstration that the bursa of Fabricius is required for the production of antibodies by B cells in newly hatched chicks (*Poultry Science*, reference 38), and Shimomura's discovery of jellyfish associated fluorescence (*Journal of Cellular and Comparative Physiology*, reference 39), which ultimately led to the discovery of green fluorescent protein in jellyfish (40). At the end of the day, what matters for a publication is content, not publication venue.

### Suggestions for avoiding the prisoner's dilemma in science

1. Although the behavior of scientists can be analyzed in the game theory problem known as the prisoner's dilemma, science offers options in addition to cooperation or defection, as scientists are not prisoners being held incommunicado. A scientist who is fearful of being scooped or sharing reagents may avoid the prisoner's dilemma by communicating with other groups. We recommend that scientists with concerns about credit reach out to their colleagues. Communication creates more options than simple cooperation or defection, which can result in the greatest benefit to all.
2. When considering whether to cooperate or defect, scientists should consider the potential benefits and detriments of their actions for the broader scientific enterprise and for humanity. Science will play an essential role as humanity confronts a looming series of existential threats including climate change, overpopulation, pandemics, and world hunger. When the benefits and detriments are considered, it is clear that any action that damages the enterprise creates a costly “tragedy of the commons,” which should strongly encourage a search for a cooperative solution.
3. Finally, scientists should remember to approach any dilemma in their interactions with other scientists humanely. Science is a human endeavor, and every scientific competition involves people who are facing similar aspirations, challenges, and fears. Kant's categorical imperative compels scientists to behave in a manner in which they would like all scientists to behave, whether in medical research (41), peer reviewing (42, 43), or other cooperative activities (44).

We find it reassuring that in the most common versions of the prisoner's dilemma faced by scientists, cooperation appears to be the wisest choice, both for the individual and for the community. If scientists can see themselves as sharing in something greater than themselves, this can reduce stress and enhance job satisfaction. One might even call it “liberating.”
